# Frailty and Postoperative Complications in Older Adults With Nonmetastatic Breast Cancer

**DOI:** 10.1001/jamanetworkopen.2025.31841

**Published:** 2025-09-15

**Authors:** Eliza H. Lorentzen, Yu-Jen Chen, Claire Morton, Tari A. King, Elizabeth A. Mittendorf, Christina A. Minami

**Affiliations:** 1 Department of Surgery, Brigham and Women’s Hospital, Boston, Massachusetts; 2 Center for Surgery and Public Health, Brigham and Women’s Hospital, Boston, Massachusetts; 3 Breast Oncology Program, Dana-Farber/Brigham Cancer Center, Boston, Massachusetts

## Abstract

This cross-sectional study investigates the association between frailty and postoperative complications in oncologic breast surgery among older adults with nonmetastatic breast cancer.

## Introduction

Frailty, a condition marked by increased vulnerability to stressors, is associated with higher postoperative complication rates after major surgery,^1^ but this association in oncologic breast surgery, which includes lower-risk operations,^2^ remains ill defined. We examined the association between frailty and postoperative complications in older adults with nonmetastatic breast cancer.

## Methods

This cross-sectional study followed the STROBE reporting guideline. The Mass General Brigham Institutional Review Board deemed this study exempt from review and consent because this was a deidentified dataset.

Women aged 70 years and older undergoing surgery for nonmetastatic breast cancer September 2013 to September 2015 were identified in SEER-Medicare. We excluded patients who had a prior history of breast cancer, received neoadjuvant chemotherapy or radiation, received reconstructive surgery, had unknown surgery type, or had noncontinuous Medicare Parts A and B coverage 1 year prior and after diagnosis. Frailty was defined as a score greater than 0.2 on a validated claims-based index^3^ that incorporates 93 variables defined by Current Procedural Terminology procedure codes, *International Classification of Diseases, Ninth Revision *(*ICD-9*) diagnosis, and Healthcare Common Procedure Coding System codes present for a patient in the 12 months prior to diagnosis. We identified local and systemic complications within 30 days of the index procedure using claims data. Local complications included surgical site infection, seroma, hematoma, and wound complications.^4^ Systemic complications included sepsis, pneumonia, myocardial infarction, deep vein thrombosis, and pulmonary embolism.^5^

Multivariable logistic regression models were performed, adjusting for a priori chosen covariates, including age, race and ethnicity as coded in SEER, socioeconomic status (defined by Yost score), urban vs rural status, and breast surgery type (lumpectomy vs mastectomy). A 2-sided *P* < .05 was considered statistically significant. Analyses were conducted from July 2024 to March 2025 using SAS software version 9.4 (SAS Institute).

## Results

Among 6963 women (2214 aged 70-74 years [31.8%]; 505 Black [7.3%], 389 Hispanic White [5.6%], 5550 non-Hispanic White [79.7%], and 519 other [7.5%]), 439 women (6.3%) had frailty. A greater proportion of patients with vs without frailty were aged 85 years or older (19.3% vs 13.4%), had a Charlson Comorbidity Index score of 2 or greater (64.7% vs 16.5%), and had undergone a mastectomy (56.0% vs 49.3%) (all *P* < .001). Overall, 968 patients (13.9%) had complications (systemic: 2.8%; local: 11.1%) (Table 1). Rates of overall complications by procedure type ranged from 571 patients undergoing lumpectomy only (8.2%) to 1170 patients undergoing mastectomy with axillary surgery (16.8%). Patients with vs without frailty had higher systemic (4.8% vs 2.7%; *P* = .009) and local (15.1% vs 10.8%; *P* = .006) complication rates. In adjusted analyses, frailty was associated with a higher likelihood of local (odds ratio, 1.48; 95% CI, 1.12-1.96) and systemic (odds ratio, 1.76; 95% CI, 1.10-2.81) complications, but chronological age was not (Table 2). Mastectomy and axillary surgery were independently associated with higher rates of local but not systemic complications.

**Table 1.** Local and Systemic Complication Rates

30-d Postoperative complication	Patients, No. (%) (N = 6963)		*P* value
	Without frailty (n = 6525 [93.7%])	With frailty (n = 438 [6.3%])	
Systemic^a^	174 (2.7)	21 (4.8)	.009
Local	707 (10.8)	66 (15.1)	.006
Hematoma or hemorrhage	238 (3.7)	26 (5.9)	.02
Surgical site infection	308 (4.7)	28 (6.4)	.11
Seroma	260 (4.0)	25 (5.7)	.08
Other wound complication (wound dehiscence or need for drainage)	359 (5.5)	38 (8.7)	.006

^a^ Systemic complication by type is not shown in compliance with Centers for Medicare & Medicaid Services cell size–suppression policy.

**Table 2.** Adjusted Logistic Regression for Odds of Local and Systemic Complications

Characteristic	Complication, OR (95% CI)	
	Local	Systemic
Age, y		
70-74	1 [Reference]	1 [Reference]
75-79	0.97 (0.80-1.17)	1.05 (0.72-1.54)
80-84	1.02 (0.83-1.25)	1.33 (0.90-1.96)
≥85	0.82 (0.64-1.05)	1.21 (0.78-1.88)
Frailty		
No	1 [Reference]	1 [Reference]
Yes	1.48 (1.12-1.96)	1.76 (1.10-2.81)
Race and ethnicity		
African American or Black	1 [Reference]	1 [Reference]
Hispanic or Latinx White	0.94 (0.67-1.32)	0.80 (0.40-1.59)
Non–Hispanic or Latinx White	0.97 (0.72-1.32)	1.58 (0.97-2.57)
Other or unknown^a^	1.14 (0.87-1.51)	0.65 (0.33-1.28)
SES, Yost score		
Group 1 (lowest SES))	1 [Reference]	1 [Reference]
Group 2	1.20 (0.92-1.56)	1.00 (0.61-1.65)
Group 3	0.97 (0.73-1.27)	1.00 (0.60-1.66)
Group 4	1.04 (0.79-1.38)	1.28 (0.78-2.11)
Group 5 (highest SES)	1.23 (0.94-1.62)	0.88 (0.52-1.48)
Urban vs rural status		
Urban	1 [Reference]	1 [Reference]
Rural	1.14 (0.93-1.41)	1.16 (0.79-1.72)
Surgery type		
Lumpectomy	1 [Reference]	1 [Reference]
Mastectomy	1.75 (1.48-2.08)	1.03 (0.75-1.41)
Axillary surgery^b^		
No	1 [Reference]	1 [Reference]
Yes (SLNB or ALND)	1.41 (1.12-1.77)	1.08 (0.74-1.58)

Abbreviations: ALND: axillary lymph node dissection; SES, socioeconomic status; SLNB, sentinel lymph node biopsy; OR, odds ratio.

^a^ Other or unknown race and ethnicity: patients coded as American Indian, Asian Indian, Chamorran, Chinese, Fiji Islander, Filipino, Guamanian, Hawaiian, Hmong Kampuchean, Japanese, Korean, Laotian, Melanesian, Micronesian, New Guinean, other Asian not otherwise specified, Pacific Islander not otherwise specified, Pakistani, Polynesian, Samoan, Tahitian, Thai, Tongan, Vietnamese, other, or unknown in Surveillance, Epidemiology, and End Results (SEER). Analyses were adjusted for race and ethnicity given the association of certain racial and ethnic groups with prevalence of postoperative complications.

^b^ Axillary surgery includes SLNB and ALND.

## Discussion

Previous studies have documented low rates of systemic complications (mastectomy: 2.9% and lumpectomy: 1.4%) in older adults undergoing breast surgery,^6^ with estimates of local complications ranging from 14.7% to 50.6% for lumpectomy and 22.7% to 49.0% for mastectomy.^4,6^ While the overall rate of individual complications in this cross-sectional study was relatively low, slightly higher rates of hematoma or hemorrhage and wound complications in patients with frailty may be secondary to drivers of frailty (eg, comorbidities requiring anticoagulation or representing risk factors associated with complications) or physical manifestations of frailty (eg, impaired wound-healing capacity). Significantly higher summed complication rates in patients with frailty should be a factor in surgical decision-making and prompt evaluation and activation of support systems necessary to address complications in a timely manner. Patients with frailty traveling long distances, with little social support, or with low health literacy may require more robust postoperative support and navigation from clinicians or at-home services.

Study limitations include reliance on coding in SEER-Medicare data and a lack of a criterion standard to determine clinically significant frailty status and detail regarding factors associated with complication risk and clinically meaningful wound complications. Our findings suggest that frailty and not chronological age alone should be considered in surgical decision-making and planning.

## Notes

Supplement.
Data Sharing Statement
