# Supplementary material for: Frailty and Postoperative Complications in Older Adults With Nonmetastatic Breast Cancer
Source: JAMA Netw Open. 2025 Sep 15;8(9):e2531841. doi: 10.1001/jamanetworkopen.2025.31841 (PMC12439052; doi:10.1001/jamanetworkopen.2025.31841)
Supplement: Supplement. — Data Sharing Statement [file jamanetwopen-e2531841-s001.pdf]

Data Sharing Statement

Lorentzen. Frailty and Postoperative Complications in Older Adults With Nonmetastatic Breast Cancer. *JAMA Netw Open*. Published online September 15, 2025. doi:10.1001/jamanetworkopen.2025.31841

Data
Data available: No

Additional Information
Explanation for why data not available: SEER-Medicare data are publicly available to researchers
